# Artificial intelligence-based screening of phytochemicals for targeted cancer therapy

**DOI:** 10.1007/s13659-026-00599-y

**Published:** 2026-04-21

**Authors:** Livia Ramos Santiago, Estéfani Alves Asevedo, Maria Eduarda Jeunon de Oliveira, Karen Cota Pereira, Maria Fernanda da Silva Trindade, Ana Gabriela Silva Oliveira, Marina Andrade Rocha, Sojin Kang, Amama Rani, Moon Nyeo Park, Michel William Tan, Rony Abdi Syahputra, Bonglee Kim, Rosy Iara Maciel de Azambuja Ribeiro

**Affiliations:** 1 Department of Experimental Pathology, Federal University of São João del-Rei, Sebastião Gonçalves Coelho Street, 400-Chanadour, Divinópolis, MG 35501-296 Brazil; 2 Department of Pathology, Kyung Hee University, Seoul, Republic of Korea; 3 Department of Pharmacology, Universitas Sumatera Utara, Sumatera Utara Medan, Indonesia

**Keywords:** Phytochemicals, Artificial intelligence, Machine learning, Deep learning

## Abstract

Cancer remains one of the leading causes of death worldwide and continues to pose a serious public health challenge. The limited success of many current treatments—often due to toxicity, poor selectivity, and the development of drug resistance—highlights the need for new and more effective therapeutic options. Phytochemicals have emerged as a valuable source of anticancer agents, offering rich structural diversity and a wide range of biological activities. However, identifying promising compounds from the vast chemical space of natural products remains difficult using conventional screening methods, which are typically slow, costly, and inefficient. In recent years, artificial intelligence (AI) has begun to transform phytochemical-based drug discovery. Machine learning and deep learning approaches are now used to support key steps in the discovery process, including metabolite identification, virtual screening, target prediction, and toxicity assessment. By integrating chemical, biological, and multi-omics data, AI enables a more systematic and data-driven exploration of natural product diversity. Despite these advances, challenges persist, particularly the scarcity of high-quality experimental data, the structural complexity of phytochemicals, and their limited representation in public databases. This review critically examines current AI applications in phytochemical-based anticancer drug discovery and discusses emerging strategies aimed at overcoming these limitations. Overall, AI-driven phytochemical screening represents a promising path toward accelerating the development of next-generation cancer therapies.

**Graphical Abstract:**

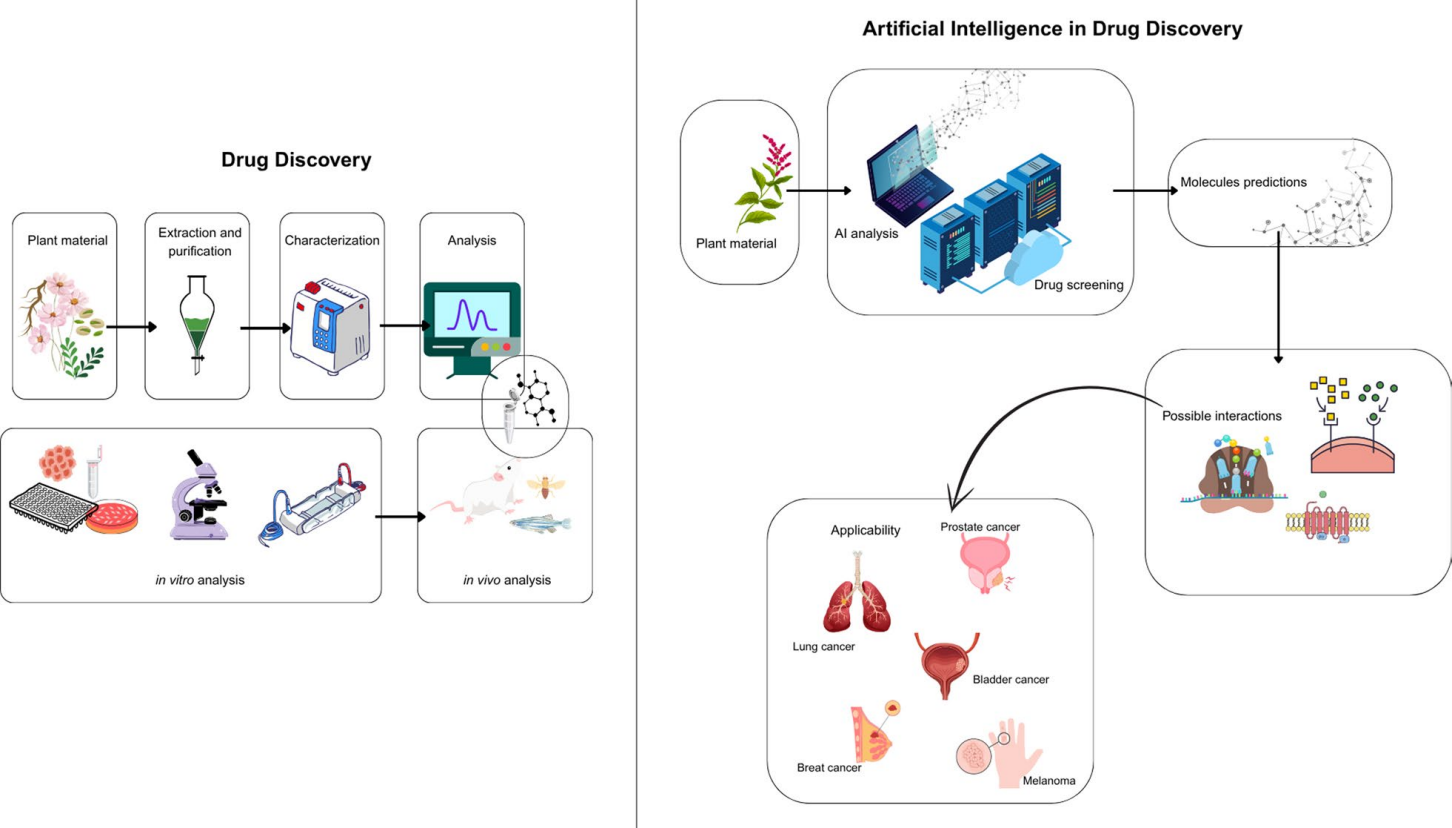

## Introduction

Cancer remains one of the leading causes of death globally, accounting for approximately one in every six deaths. Despite advances in conventional therapies such as surgery, radiotherapy and chemotherapy, limitations including severe side effects, limited selectivity, and growing drug resistance continue to hamper treatment success [1, 2]. These limitations continue to drive the search for alternative and complementary therapeutic strategies that can improve efficacy while minimizing toxicity.

In this context, phytochemicals have emerged as an attractive source of novel anticancer agents. These naturally occurring bioactive compounds, derived mainly from medicinal plants and dietary sources, exhibit diverse mechanisms of action and have been shown to enhance the therapeutic efficacy of conventional chemotherapeutic agents [3]. Due to their structural diversity and broad pharmacological activities, many phytochemicals exhibit favorable attributes such as low toxicity, biocompatibility and dual therapeutic– nutritional benefit [4]. Compared with many synthetic drugs, phytochemicals are often associated with improved safety profiles and a reduced propensity for the development of multidrug resistance [5]. Notable plant-derived anticancer drugs approved by the Food and Drug Administration (FDA) are Paclitaxel, isolated from the extract of *Taxus brevifolia*, Camptothecin, isolated from the extract of *Camptotheca acuminata Decne*, and the alkaloids vinblastine, vincristine, vindesine and vinorelbine, isolated from the extract of *Catharanthus roseus* [6–8]. Nevertheless, despite the vast chemical diversity of the plant kingdom, the systematic discovery of anticancer phytochemicals remains slow, largely due to the limitations of traditional experimental screening approaches, which are time-consuming, costly, and poorly suited for large-scale exploration [9].

In recent years, artificial intelligence (AI) has emerged as a powerful enabling technology capable of addressing these challenges in natural product–based drug discovery. AI-driven computational methods allow for the high-throughput analysis of complex chemical and biological datasets, supporting tasks such as virtual screening, target prediction, activity modeling, and lead optimization for phytochemicals with anticancer potential [9]. Importantly, phytochemical research poses unique computational challenges, including high structural complexity, limited annotated datasets, and chemical features that deviate from typical drug-like space, which often reduce the effectiveness of conventional modeling approaches. AI and machine-learning techniques are particularly well suited to overcoming these barriers by learning non-linear patterns from heterogeneous data and enabling predictive modeling even in data-constrained settings. Beyond reducing costs and development time, AI-driven methods hold the potential to revolutionize precision oncology by enabling the design of therapies that target specific cancer pathways with greater accuracy [10].

Although numerous AI tools and platforms have been developed for drug discovery, existing literature often presents these methods in a fragmented manner, with limited critical comparison, performance evaluation, or guidance on tool selection for specific phytochemical screening scenarios. Moreover, many available reviews either focus broadly on AI in drug discovery or on natural products without systematically integrating both perspectives in the context of anticancer research. To address these gaps, this review aims to provide a structured and critical overview of AI applications in phytochemical-based anticancer drug discovery, with emphasis on methodological principles, comparative strengths and limitations of existing tools, representative case studies, and practical considerations for tool selection across different research objectives. By doing so, we seek to offer not only a summary of current advances but also a decision-oriented framework to guide future research in this rapidly evolving field.

## Anticancer phytochemicals and current limitations to their discovery

Natural products are characterized by presenting scaffolding diversity and structural complexity. Compared with most synthetic small molecules, phytochemicals often exhibit higher molecular weights, increased rigidity, and a greater number of stereocenters and functional groups, features that contribute to their rich biological activity but also complicate conventional drug discovery workflows. These properties allow natural products to serve as valuable scaffolds for drug discovery, even for compounds that fall outside Lipinski’s Rule of Five. Consistent with this trend, the average molecular weight of newly approved drugs has increased in recent years, highlighting a gradual shift away from strict adherence to traditional drug-likeness criteria [11]. Despite this, natural products continue to be the foundation of most approved anticancer drugs, supported by resources such as the National Cancer Institute's Natural Products Repository, comprising more than 180,000 extracts.

In general, after identifying bioactive extracts, the next steps involve isolating individual components and characterizing their chemical structures. However, this process is often labor-intensive and technically challenging, particularly when dealing with complex mixtures and low-abundance active compounds. Practical challenges associated with handling natural products, including difficulties in sample reconstitution, dilution, liquid transfer, variability, viscosity and precipitation, all of which can limit reproducibility and scalability in high-throughput screening campaigns [12]. These limitations represent a major bottleneck in the systematic discovery and optimization of anticancer phytochemicals.

One promising approach to overcoming drug resistance in tumor cells involves the use of natural products as adjuvants. Numerous studies have demonstrated that natural compounds can resensitize cancer cells to conventional chemotherapeutics by modulating multiple resistance-associated pathways [12]. For example, treatment with the phytochemical curcumin combined with docetaxel resulted in a downregulation of drug resistance genes in MCF-7 human breast cancer cells [13]. Another example is that *Myrciaria tenella* extracts caused cell death in human cercical adenocarcinoma (HeLa) and human gastric adenocarcinoma (SFA) cell lines. In addition, the extracts did not show cytotoxicity in the 3T3 non-tumor cell line [14]. Such findings support the growing interest in phytochemicals as multitarget, potentially less toxic alternatives or complements to conventional single-target chemotherapeutic agents [15].

Natural compounds can interfere with essential cellular processes such as DNA replication, topoisomerase functioning, and microtubule dynamics, encouraging their broad investigation in preclinical and clinical studies. Among the drugs approved by the Food and Drug Administration (FDA), Camptosar, derived from the plant *Camptotheca acuminata*, used in the treatment of metastatic colorectal cancer; Etoposide, obtained from the podophyllotoxin present in *Podophyllum peltatum*, used for lung cancer and lymphomas; and Taxol, isolated from the bark of *Taxus brevifolia*, widely used in the treatment of non-small cell lung cancer, neuroblastoma, and Wilms' tumor. In addition to plant-derived agents, marine- and microorganism-derived drugs such as Halaven, Cytarabine, and Synribo further illustrate the broad diversity of natural sources contributing to modern oncology**.** Table 1 summarizes the major FDA-approved natural product-derived anticancer drugs, presenting their respective sources of natural products, the indications for cancers to be treated, mechanisms of action, and bibliographic references.

**Table 1:** List of FDA-approved natural product-derived anticancer drugs

Commercial name	Source (Natural products)	Specific cancer suppressed	Mechanism of action	Refs.
Camptosar	Extract of *Camptotheca acuminate*	Metastatic colorectal cancer	SN-38 acts as a topoisomerase I inhibitor. It binds to the DNA-topoisomerase I complex, preventing the religation of DNA single-strand breaks. The accumulation of DNA breaks during replication, interrupting the cell cycle and inducing apoptosis s	[16]
Cytarabine	Spongouridine nucleoside from the sponge *Tethya crypta* (*Cryptotethya crypta*)	Hematologic neoplasm	It is a deoxycytidine analog that acts on cells during DNA synthesis, specifically killing them in the S phase of the cell cycle, and can also block the transition of cells from the G1 phase to the S phase	[17]
Etoposide	Derives from podophyllotoxin, a toxin found in the *Podophyllum peltatum*	Lung cancer, germ cell tumors, and refractory lymphomas	Etoposide acts by inhibiting topoisomerase II, causing double-strand breaks in DNA and forming a complex by binding to the DNA-topoisomerase II, causing an impediment to the religation of the double-strand breaks in DNA. As a result, there is cell cycle arrest and induction of apoptosis	[18]
Halaven	Isolated from the marine sponge *Halichondria okadai*	Metastatic breast cancer	Binds with high specificity to the ends of microtubules, inhibiting polymerization without affecting depolymerization, causing disorganization of the mitotic spindle, arrest in the G2/M phase of the cell cycle, prolonged mitotic blockade, and induction of apoptosis	[19]
NAVELBINE	Derived from *Vinca rosea*	Non-small cell lung cancer	Inhibition of microtubule formation in mitotic spindle, resulting in an arrest of dividing cells at the metaphase stage	[20]
Synribo	Vincristine Sulfate Injection; extract from leaves of *Cephalotaxus*; alkaloid from *Vinca rosea*	Chronic myeloid leukemia; Leukemia, Hodgkin’s disease, non–Hodgkin’s lymphomas, rhabdomyosarcoma, neuroblastoma, Wilms’ tumor	Inhibits protein synthesis and microtubule formation in mitotic spindle, arresting cells at metaphase	[21]
Taxol	Originally isolated from the bark of *Taxus brevifolia*	Non-small cell lung cancer	Promotes the assembly of microtubules from tubulin dimers and stabilizes microtubules by preventing depolymerization	[22]

Despite their proven therapeutic value, the development of natural product–based anticancer drugs remains constrained by several fundamental limitations. One major challenge is the availability and sustainable supply of source organisms. Many microorganisms that produce bioactive metabolites are difficult to cultivate outside their natural habitats, which can compromise the continuous production of these compounds. To address this issue, alternative strategies such as in situ analysis, synthetic induction of biosynthetic pathways, and heterologous expression of biosynthetic gene clusters in model organisms have been explored [23]. In addition, crude extracts often contain reduced concentrations of the active ingredients, which makes their isolation and characterization difficult.

Another significant challenge concerns the distribution and bioavailability of natural compounds. Many natural compounds undergo extensive first-pass hepatic metabolism, leading to reduced plasma concentrations and diminished therapeutic efficacy [24]. Often, high doses are required to achieve clinically relevant effects. In this context, the use of lipid carrier systems has shown promise. These carriers, owing to their biocompatibility, biodegradability, and capacity for controlled release, offer promising strategies to enhance the stability, absorption, and bioavailability of phytochemicals [25].

Beyond pharmacokinetic constraints, the clinical development of herbal medicines faces additional challenges, including poor aqueous solubility, chemical instability, and specific regulatory requirements. For example, classification as a Traditional Herbal Product (THP) often requires evidence of long-term traditional use, which may not align with modern drug development paradigms. To overcome these barriers, various pharmaceutical strategies, such as nanoparticles, liposomes and hydrogels, have been investigated with the aim of overcoming these limitations, promoting greater stability, solubility and sustained release. However, despite encouraging preclinical findings, robust and well-designed preclinical and clinical investigations remain essential to fully establish the efficacy and safety and translational viability of these approaches [26].

Collectively, these challenges underscore the need for innovative, scalable, and integrative strategies to accelerate the discovery and development of anticancer phytochemicals—an area where computational and AI-driven approaches offer considerable promise, as discussed in subsequent sections.

## Artificial intelligence in drug discovery and screening

The development of new drugs is increasingly benefiting from artificial intelligence (AI) and big data, which allow for faster and more efficient processes, reduced reliance on animal testing, and minimized human bias. These technologies also enable more accurate analysis and handling of complex chemical and biological data compared with traditional manual approaches. A notable example of this is QSAR (quantitative structure–activity relationship), a computational method used to predict the biological activity of chemical compounds. AI-enhanced QSAR models improve predictive performance by capturing complex, non-linear relationships between molecular descriptors and biological activity. Such models are now applied across multiple stages of drug discovery, from target identification and validation to guiding clinical trial design [27].

Artificial Intelligence (AI) is rapidly reshaping the landscape of drug discovery and development, addressing solutions to long-standing challenges such as inefficiency, high costs, and ethical concerns, including the need to reduce animal testing and mitigate human bias.

Advanced AI techniques, including machine learning (ML), deep learning (DL), and natural language processing (NLP), are now widely adopted to predict molecular properties, identify druggable targets, and optimize lead compounds with improved precision and speed.

By leveraging vast and diverse datasets, ranging from genomics and chemical structures to clinical records, AI models can uncover hidden patterns and generate actionable hypotheses far more rapidly than traditional methods. A comprehensive review by Vamathevan et al. highlights how AI-driven platforms can significantly accelerate hit identification while reducing attrition during early-stage drug development [27, 28].

Furthermore, the tools allow us to identify whether natural compounds can inhibit specific targets. In cancer research, signaling pathways play a central role in therapeutic development, with the mitogen-activated protein kinase (MAPK) pathway being one of the most extensively studied. The MAPK pathway is thought to be involved in glioma development. This pathway interacts with several bioactive regulators. In carcinogenesis, dysregulation of this pathway has been associated with both therapeutic resistance and sensitivity. The MAPK signaling pathway is upregulated in glioma. To model pathway-associated prognostic risk, least absolute shrinkage and selection operator (LASSO) regression has been applied to select relevant genes and coefficients for machine-learning–based risk models. As a result, it was demonstrated that high-risk groups tend to have shorter survival times, while the low-risk group has a more dispersed representation in the analysis range, resulting in a wider survival time [29].

Zhang et al. (2019) demonstrated the use of a computational modeling strategy to evaluate the response of the HCT116 colorectal cancer cell line to cisplatin and TRAIL at the single-cell level. Their models predicted that combined cisplatin and TRAIL treatment increased the rate of cell death compared with cisplatin alone and revealed a bimodal distribution in the timing of tumor cell death. Partial least squares regression, expected forest, logistic regression, and support vector machine analysis were also employed. The study identified key determinants of drug sensitivity, including BCL family proteins, p53 regulatory feedback loops, and the processing rate of Bcl-2 homology 3 (BH3) domain proteins [30].

One of AI's most transformative applications is in virtual screening and molecular design. AI-driven algorithms now enable rapid in silico screening of millions of compounds, predicting binding affinities and interactions with target proteins at unprecedented speed. Tools such as graph neural networks (GNNs), variational autoencoders (VAEs), and reinforcement learning models are being leveraged to generate novel, drug-like molecules optimized for specific biological targets. A notable example is Exscientia, which advanced AI-designed drug candidates to clinical trials in less than one year, highlighting the translational potential of these approaches**.** As reviewed by Lavecchia (2021), deep learning models have significantly improved the efficiency and accuracy of structure-based virtual screening [31, 32].

AI also plays a critical role in predicting ADMET properties, Absorption, Distribution, Metabolism, Excretion, and Toxicity. Early identification of compounds with unfavorable pharmacokinetic or toxicity profiles is essential to reducing late-stage failure rates and development costs. Machine-learning models trained on experimental ADMET data help flag potentially unsafe compounds and prioritize those with desirable properties. Recent studies, including work by Wu et al., demonstrate that AI-based toxicity prediction, particularly using ensemble and deep learning approaches—often outperforms traditional QSAR models across diverse chemical spaces [33, 34].

Despite the promising advances, the integration of AI into drug discovery pipelines still faces several challenges. Limitations related to data quality, dataset bias, biological relevance, and model interpretability can hinder the reliability and translational impact of AI-driven predictions. Many AI models operate as “black boxes”, lacking transparency, which complicates experimental validation and regulatory acceptance. As highlighted by Walters & Barzilay [35], the development of explainable AI (XAI) frameworks is critical for building trust and facilitating adoption in clinical and regulatory settings. Looking ahead, the successful application of AI in drug discovery will depend on continued collaboration between computational scientists, chemists, and biologists. Bridging these disciplines will be key to harnessing AI’s full potential, enabling smarter, faster, and more ethical drug development.

## AI tools in phytochemical for drug discovery

**Fig. 1:**
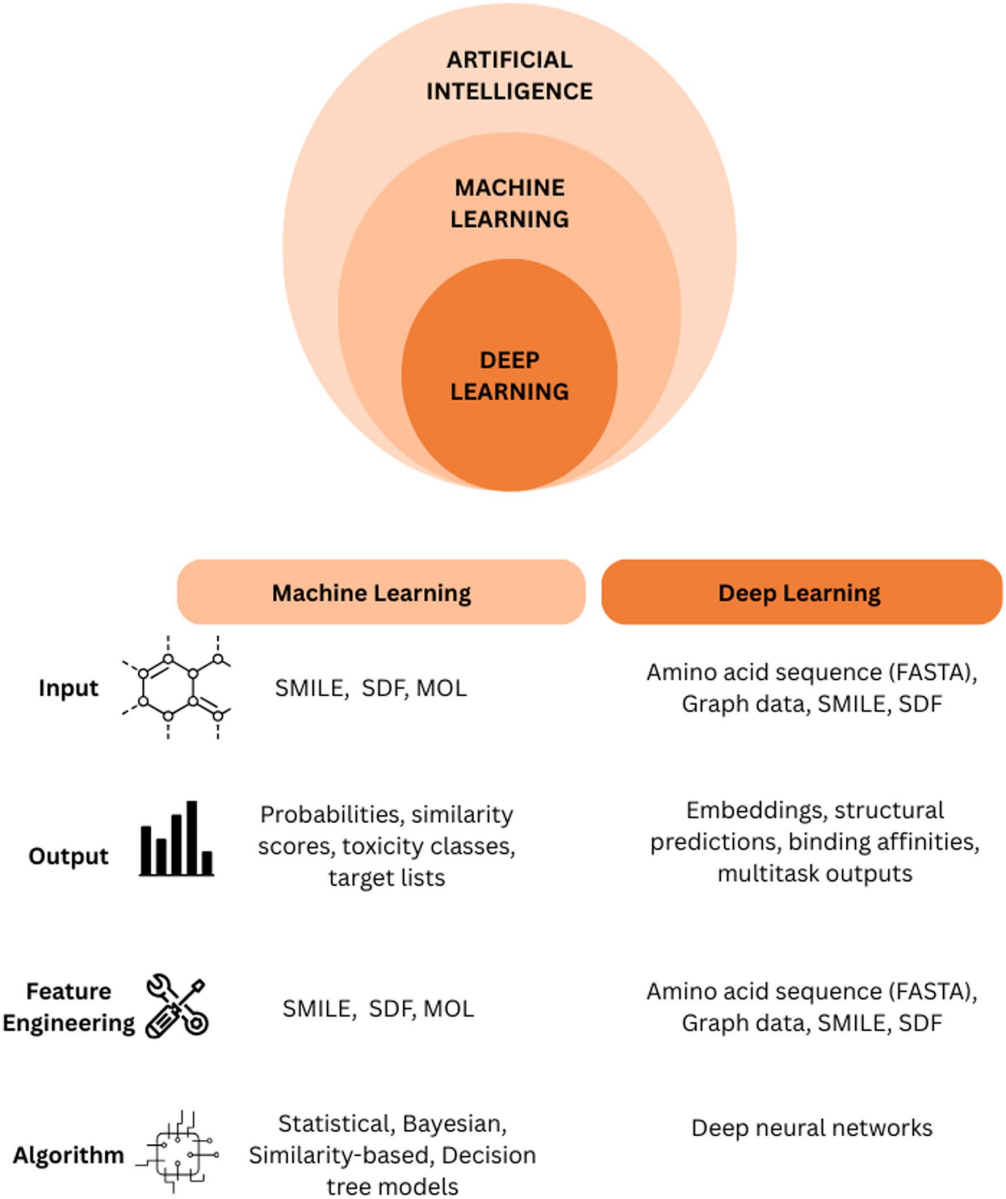
Comparison between Machine Learning and Deep Learning approaches in artificial intelligence for molecular and biological data. The concentric diagram illustrates the hierarchical relationship between Artificial Intelligence (AI), Machine Learning (ML), and Deep Learning (DL). The table compares ML and DL based on four aspects: input data, output predictions, feature engineering, and algorithms.

AI can support various stages of the drug discovery process, for example, by analyzing spectrometry data, performing automated searches across chemical and biological databases, and predicting compound identities. Once candidate compounds are identified, AI-based tools can further assist in toxicity prediction, target interaction analysis, and mechanism-of-action elucidation, which are critical steps in both anticancer drug development and translational research (Fig. 1).

Machine learning (ML) models, including supervised, unsupervised, and semi-supervised approaches, have been widely applied and analyze data related to natural compounds with potential anticancer activity, providing insights and predicting properties of novel compounds [36, 37]. ML typically operates based on labeled or structured data, which determines the nature of the learning process as summarized in Table 2. A more specialized subset of ML is deep learning (DL), which employs multi-layered neural networks capable of automatically extracting informative features from raw molecular or biological data. This hierarchical representation allows DL models to capture complex, non-linear relationships that are often inaccessible to traditional ML approaches. As a result, DL methods have shown particular promise in drug discovery, especially when dealing with high-dimensional biological data and structurally complex natural products [38].

**Table 2:** Machine learning and deep learning

Machine learning		
Methods	Description	Refs.
Supervised learning	Predictions are made based on a set of labeled data by modeling the relationship between a set of variables (features or predictors) and the output variable of interest	[39]
Unsupervised learning	Identify underlying dimensions, components, clusters, or trajectories within a data structure	[40]
Semi-supervised learning	Uses sparsely labeled data and a large amount of auxiliary unlabeled data often drawn from the same underlying data distribution as the labeled data	[41]
**Deep learning**		
**Methods**	**Description**	**Refs.**
Deep neural networks	Artificial neural network that is incorporated with many layers amid input layers and output layers of the network	[42]
Graph-based approaches	Table structure reconstruction steps from detected building components	[43]
Natural language processing	Applied to extract information on DDIs, as well as on drug–drug interactions, from unstructured text sources, such as the scientific literature, electronic health records, and drug labels	[36]
Text mining	Extracting structured information from unstructured text	
Generative modeling-based approaches	Learning the data’s underlying distribution with the intent of generating new samples	

Table 3 provides an overview of several ML-tools used to predict chemical properties and molecular interactions. These ligand-based approaches rely primarily on chemical similarity, molecular descriptors, or learned structure–activity relationships derived from curated bioactivity databases. DEcRyPT (Drug–Target Relationship Predictor) is based on random forest regression that uses the ChEMBL database as a training set and from there predicts continuous affinity values from molecular descriptors [44].

**Table 3:** Machine learning–based target fishing tools

Software	Strategy	Algorithm	Description	Website	Refs.
DEcRyPT	Ligand-based	Random forest (Regression)	Drug-target interactions prediction	–	[44]
PASS Online	Ligand-based	Neural network (Feed-forward, Classical ANN)	Biological activity prediction	https://www.way2drug.com/passonline/	[52]
ProTox-II	Ligand-based virtual screening	Random forest (Ensemble Methods)	Toxicity prediction	https://tox.charite.de/protox3	[53]
SEA	Ligand-based	Similarity-based method (Fingerprint comparison)	2D molecular similarity	https://sea.bkslab.org/	[54]
SPiDER	Ligand-based	Similarity-based method (Pharmacophore descriptors)	Drug Equivalence Relationships	–	[50]
STP	Ligand-based	Machine learning (k-Nearest neighbors, logistic regression, 2D/3D similarity)	2D and 3D molecular similarity	http://www.swisstargetprediction.ch	[55]

PASS is a target fishing model that predicts a spectrum of biological activity based on structural patterns using a Multilevel Neighborhoods of Atoms (MNA) substructure descriptor algorithm. For each biological activity, numerical values, Pa and Pi, are provided, corresponding to the probability of the compound exhibiting that activity or not, respectively. Pramely and Raj et al. used PASS to predict the biological activities of *Azadirachta indicia* A. Juss, for each phytoconstituent, a series of activities were listed, with their respective Pa and Pi values [45]. Kadir et al. used PASS to predict the biological activities of *Vitex negundo*, the antioxidant and antiproliferative activities were experimentally validated [46].

ProTox-II allows rapid toxicity prediction based on the structural similarity to experimentally characterized compounds combined with toxicophore-based modeling. This approach allows early-stage safety assessment and prioritization of natural compounds before experimental validation. Asevedo et al. used the model to predict the LD50 of natural compounds in a Review Paper that highlighted the pharmacological properties of *Caesalpinia sappan* [47]. Boukhira et al. used ProTox to predict toxicological endpoints such as hepatotoxicity, carcinogenicity, immunotoxicity, mutagenicity, and other activities of *Thymus broussonetii* Boiss. essential oil [48].

SEA (Similarity Ensemble Approach) is based on chemical similarity between known ligands, linking 2D similarity with statistical significance for each target using bioactivity data derived from ChEMBL. Mayr et al. evaluated potential molecular targets of known natural products using three target prediction tools, including SEA. The authors incorporated statistical calculations across the three tools to summarize the evidence, and finally, the in silico findings were experimentally confirmed [49].

SPiDER (Self-organizing map-based Prediction of Drug Equivalence Relationships) predicts molecular targets based on pharmacophoric and physicochemical descriptors, generating ranked lists of potential protein targets [50]. Rodrigues et al. used SPiDER to identify potential targets of β-lapachone and refined predictions using DEcRyPT. Experimental validation confirmed the inhibition of a tumor-associated enzyme, illustrating the complementary use of multiple AI tools in natural product research [44].

Swiss Target Prediction (STP) is a ligand-based approach combining 2D and 3D metrics with ML classifiers. Adnan et al. STP and SEA to identify molecular targets of *C. sappan* phytochemicals, linking predicted targets to genes associated with type 2 diabetes mellitus and performing protein–protein interaction network analysis [51].

Table 4 presents deep learning–based tools for predicting molecular properties, drug–target interactions, and biological activities. AlphaFold2 is a deep-learning model for protein structure prediction. Although not specific to natural products, AlphaFold2 provides near-experimental protein structures that substantially improve structure-based studies of phytochemical–target interactions [56, 57].

**Table 4:** Deep learning–based target fishing tools

Software	Strategy	Algorithm	Description	Website	Refs.
AlphaFold	Structure-based	Deep neural network (Transformer + Convolutional Neural Network)	Protein 3D structure prediction	https://alphafold.ebi.ac.uk/	[67]
ChemBERTa	Ligand-based	Transformer (BERT adapted for SMILES)	Biological activity prediction	https://deepchem.io/tutorials/transfer-learning-with-chemberta-transformers/	[68]
DeepChem	Framework	Random Forest, Support Vector Machine, Convolutional Neural Network, Graph Neural Network, Transformer, etc	Molecular property prediction, Drug-target interaction prediction, Toxicity prediction, Bioactivity prediction, etc	https://deepchem.io/	[69]
DeepNPD	Ligand-based + network-based	Ensemble de Deep Neural Networks	Synergistic anticancer potential	–	[60]
PyTorch Geometric (PyG)	Graph-based molecular modeling	Graph Neural Network	Drug-target interaction prediction, Bioactivity prediction	https://graph-neural-networks.github.io/	[61]
MolBERT	Ligand-based	Transformer (BERT adapted for SMILES)	Biological activity prediction	https://github.com/BenevolentAI/MolBERT	[70]
MT-DTI	Ligand + target-based	Transformer (Molecule Transformer)	Drug-target interaction prediction	https://github.com/deargen/mt-dti	[71]
STarFish	Ligand-based	Deep Neural Network (Multitask, Fully Connected)	Biological activity prediction	https://github.com/ntcockroft/STarFish	[66]

Jung et al. demonstrated the effectiveness of the ChemBERTa by predicting ADMET properties of DrugBank compounds, showing its utility for early-stage screening and optimization [58]. Zhou et al. compiled a library of natural compounds with therapeutic potential for Alzheimer's disease and applied DeepChem to predict protein–ligand affinity, ADMET properties, and computational ranking [59].

Cao developed DeepNPD to predict synergistic natural products acting on shared targets via distinct mechanisms. Notably, the model demonstrated robustness when trained on the HERB database, performing well despite limited data availability and high chemical complexity characteristic of natural products [60].

PyTorch Geometric (PyG) supports graph neural networks (GNNs) that represent molecules as graphs, enabling accurate modeling of atomic connectivity. When integrated with generative graph models such as GraphVAE, GraphGAN, and MolGAN, PyG facilitates the generation of optimized molecular derivatives beyond traditional fingerprint-based approaches [61]. PyTorch Geometric is a tool for Graph Neural Networks (GNN). Because it receives data structured as graphs, it is highly flexible, with considerable architectural freedom allowing the creation of custom Graph Neural Networks. In the context of drug discovery, it can predict the biological activities of new molecules. When combined with other generative graph models (GraphVAE, GraphGAN, MolGAN), it can generate molecules with optimized derivatives. The advantage is that by using graphs, the GNN captures the true structure and connectivity of atoms, something that traditional methods based on fingerprints or fixed vectors cannot fully represent [61].

MolBERT is a SMILES-based language model trained to generate molecular embeddings for bioactivity prediction. Norinder demonstrated that MolBERT outperformed conventional methods for toxicity prediction, achieving higher sensitivity, specificity, and balanced accuracy [62]. Related models, including SMILES-BERT and MolCLR, have also shown strong performance in predicting physicochemical properties such as lipophilicity and solubility [63]. Li and Jiang further reported that MolBERT outperformed other models on standard benchmarks, including BBBP, Tox21, SIDER, and ClinTox datasets [64]. MolBERT is a SMILES-based language model that generates molecular representations (embeddings) and is trained to represent molecules and predict bioactive properties. Norinder compared MolBERT with traditional approaches to predict toxicity endpoints, and the tool performed better with high sensitivity, specificity, and balanced accuracy [62]. In addition to MolBERT, other models such as SMILES-BERT and MolCLR are also highlighted for showing high accuracy in predicting physicochemical properties, such as lipophilicity, solubility and distribution coefficient [63]. Li and Jiang also demonstrated that MolBERT outperformed other tested models for predicting four standard datasets: Blood–Brain Barrier Penetration (BBBP), toxicity (Tox21), Side Effect Resource (SIDER), and clinical toxicity (ClinTox) [64].

Lee et al. applied MT-DTI to identify natural compounds targeting the TRPV ion channel, predicting the flavonoid troxerutin as a promising antagonist. These predictions were subsequently validated through molecular docking, in vitro assays, and a small-scale clinical trial [65]. Lee et al. used MT-DTI to identify potential natural product molecules with antagonistic activity on the TRPV ion channel. The flavonoid troxerutin was predicted to be a promising antagonist, which was corroborated by molecular docking, in vitro assays, and a small-scale clinical trial [65].

The application of ML and DL tools to natural product target fixhing faces two major challenges: limited representation of phytochemicals in public databases and high structural complexity. Consequently, models trained predominantly on synthetic compounds may exhibit reduced accuracy when applied to natural products. To address this challenge, Cockroft et al. developed a model-stacking approach, combining multiple algorithms with a meta-classifier for reverse prediction Although trained on synthetic compounds from ChEMBL, STarFish was evaluated using a natural product benchmark and demonstrated improved prediction performance for nuclear receptors, enzymes, and GPCRs [66].

Table 5 presents selection of bioinformatics tools designed in the analysis of mass spectrometry. These tools use AI or advanced computational algorithms to annotate fragmentation spectra, assign molecular formulas, and suggest candidate compound identities, thereby supporting large-scale metabolomic and phytochemical studies. In addition to compound identification, these tools are commonly used for data sharing and statistical analyses, allowing for the quantification and comparison of compounds across samples.

**Table 5:** Bioinformatics tools specialized in the analysis of mass spectrometry data

Function	Software	Details	Website	Refs.
Annotation of molecular fragments	MS2LDA	Decompose sets of molecular fragmentation	ms2lda.org	[73]
	MAGMa	Automatic chemical annotation of mass spectrometry data	https://research-software-directory.org/ software/magma	[74]
Compound identification	SIRIUS 4	Identify metabolites using single and tandem mass spectrometry	https://bio.informatik.uni-jena.de/software/ sirius-4–0-1/	[75]
	CFM-ID	Predict, annotate and interpret tandem mass (MS/MS) spectra of small molecules	https://cfmid.wishartlab.com	[76]
	MetFrag	Identification of the structure of molecules by annotation of high-precision tandem mass spectra of metabolites	https://msbi.ipb-halle.de/MetFrag/	[77]
	MS-FINDER	Annotation program that supports EI-MS (GC/MS) and MS/MS spectral mining	https://systemsomicslab.github.io/compms/ msfinder/main.html	[78]
Spectrum sharing and fragmentation	GNPS	Share raw, processed, or annotated fragmentation mass spectrometry data (MS/MS)	https://gnps.ucsd.edu/ProteoSAFe/static/ gnps-splash.jsp	[79]
Statistical analysis and interpretation	MetaboAnalyst	Metabolomic data analysis and interpretation of NMR and MS data	https://www.metaboanalyst.ca/	[80]
Visualization and quantification	MZmine 2	Visualization and basic statistics analyses	https://mzio.io/	[81]
	XCMS	Used to process LC/MS-based metabolomic data	https://xcmsonline.scripps.edu/landing_ page.php?pgcontent = mainPage	[82]

Such tools frequently interface with plant-specific and metabolomic databases that provide molecular, genomic, taxonomic, and phylogenetic context, including HMDB, MassBank, METLIN, GNPS, ReSpect, and MoNA [72]. For proteomics, UniProt, PRIDE, PeptideAtlas, and Mascot support peptide and protein identification, while lipidomics resources such as LIPID MAPS and LipidBlast facilitate lipid annotation. Databases including DrugBank, T3DB, and PubChem further support the annotation of xenobiotics and bioactive compounds.

Studies in the literature have shown that the use of these tools is promising. Studies by Mu et al. evaluated the primary targets that herbs present in the Sargentodoxa cuneata formulation commonly used in traditional Chinese medicine, and the results obtained in silico corroborated the results obtained in vitro [83]. The same tools were developed to elucidate the mechanisms of action of Bazhen Decoction [84] and Quercetin [85], and both were validated in vitro using different tumor cell lines.

AI tools have also enabled the identification of compound–target affinities and guided molecular optimization. For example, indirubin was identified as a key inhibitor of RPS20 in colorectal cancer, and AI-guided optimization led to the design of 20 derivatives with enhanced activity [86]. Another study screened 18 varieties of date pits using AI methods, revealing substantial variability in bioactive composition and functional properties with prediction accuracies reaching 92.57% [87]. Collectively, these studies demonstrate the ability of AI to integrate bioactivity, bioavailability, and tissue-specific information into phytochemical research pipelines [88].

The tools and studies discussed illustrate that AI enables large-scale bioactivity prediction, toxicity assessment, structural elucidation, and data-driven discovery. Ligand-based platforms such as PASS Online, ProTox-II, SEA, SPiDER, STP, and DEcRyPT generally exhibit good predictive performance on benchmark datasets (AUC ≈ 0.80–0.90), but their accuracy declines for structurally novel phytochemicals due to limited chemical space coverage and increased false-positive rates[53, 54, 89]. Deep learning approaches, including ChemBERTa, MolBERT, DeepChem, DeepNPD, PyG, MT-DTI, and STarFish, consistently outperform traditional fingerprint-based models in QSAR and target prediction benchmarks, achieving up to 10% improvement in predictive metrics, albeit with higher computational cost and data requirements [68, 70, 90]. AlphaFold has further revolutionized protein structure prediction with near-experimental accuracy, indirectly supporting phytochemical research through improved protein–ligand interaction modeling [91]. For metabolomics, GNPS, SIRIUS 4, CFM-ID, MetFrag, and MS-FINDER demonstrate high annotation accuracy for high-resolution MS/MS data, while MS2LDA and MAGMa provide complementary substructure-level insights [75, 76, 79, 92]. Despite these advances, there remains a strong need for more robust, interpretable, and natural-product-specific AI tools, particularly for poorly characterized or novel phytochemicals [93].

## Proposed AI-driven framework for phytochemical discovery

Artificial intelligence (AI) has increasingly been applied in drug discovery to integrate machine learning, molecular modeling, and large-scale biological data analysis, thereby enabling faster and more cost-effective identification of bioactive compounds [94, 95]. However, most AI-driven discovery pipelines have been created and verified using datasets that are primarily composed of synthetic small molecules, which restricts how well they work with natural products and phytochemicals that have more complex structures, extensive stereochemistry, and frequent multi-target pharmacological effects [11, 96]. Numerous studies have demonstrated that traditional molecular representations, drug-likeness filters, and single-target screening methods frequently fail to adequately include the distinctive chemical space and biological properties of plant-derived compounds [97]. As a result, AI models optimized for synthetic libraries often show reduced generalizability when applied to phytochemicals. There is an increasing agreement in the literature that phytochemical discovery necessitates AI frameworks particularly tailored to incorporate natural product–focused data, multi-target bioactivity prediction, and experimental feedback mechanisms [98, 99].

**Fig. 2:**
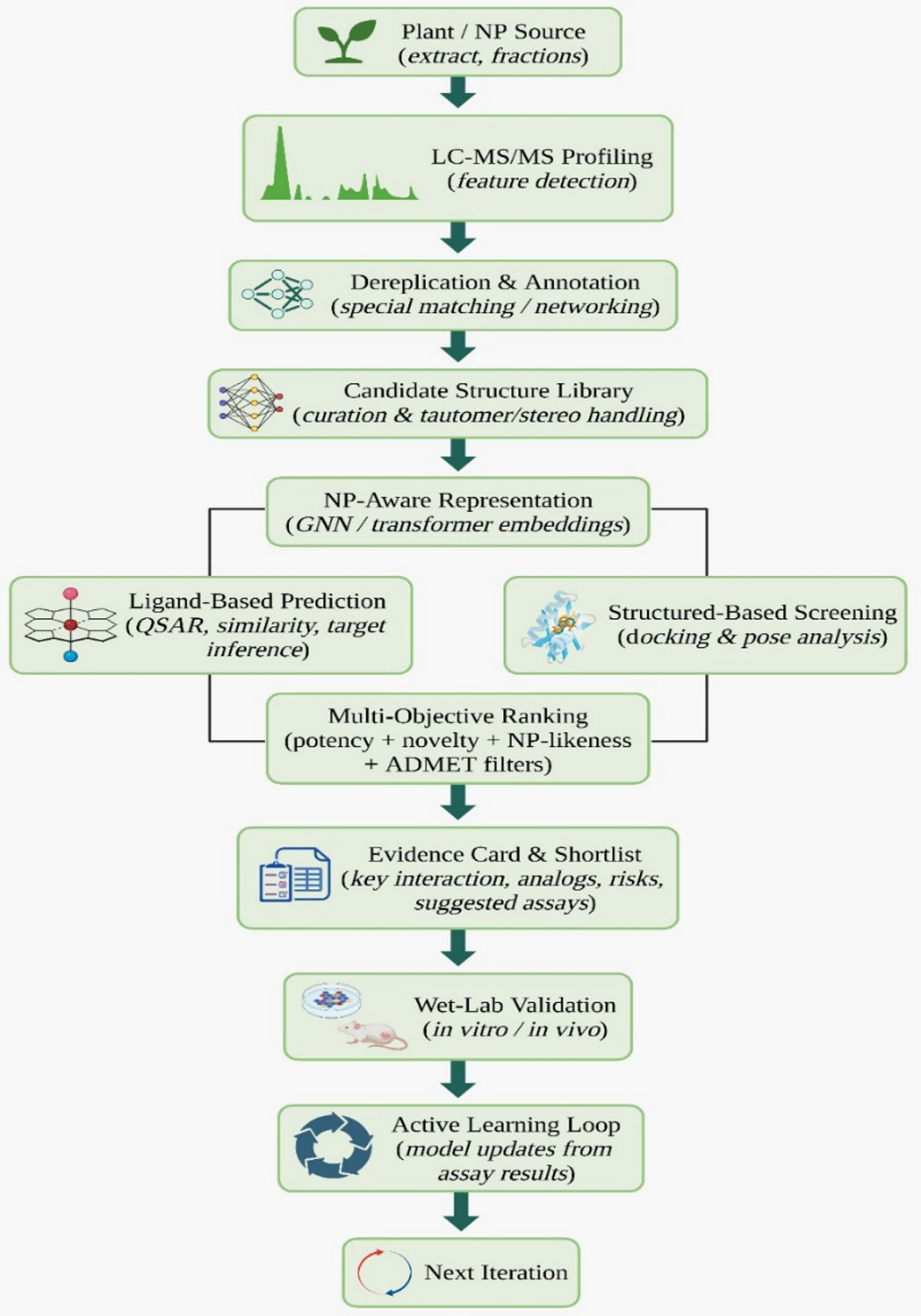
Artificial intelligence-based workflow for natural product drug discovery**.** The pipeline integrates LC MS MS metabolite profiling, compound dereplication and annotation, AI based molecular representation, ligand-based prediction, structure based virtual screening, and multi objective ranking to prioritize candidates for experimental validation and iterative model refinement

This section describes NP-ScreenR, an AI-driven framework that integrates natural product knowledge, metabolomics, and multi-level computational screening to systematically prioritize plant-derived anticancer phytochemicals (Fig. 2) [100]. NP-ScreenR is designed as a modular, decision-guided pipeline in which each stage addresses a specific limitation of conventional natural product discovery workflows identified in earlier sections.

### Plant/NP source

Plant-derived natural products remain central to cancer therapy, with approximately 60% of approved anticancer drugs derived from or inspired by phytochemicals. Consequently, selecting promising plant sources is a crucial initial step. AI approaches now integrate ethnopharmacological knowledge with large chemical and bioactivity databases to prioritize plants with high anticancer potential. By learning patterns from historical usage, chemical composition, and genomic information, machine learning models can rank plant species or extracts according to predicted therapeutic relevance. This integration streamlines the traditionally slow discovery process and provides a focused, data-driven foundation for NP-ScreenR, directing subsequent analyses toward high-value natural sources [101].

### LC–MS/MS profiling

After a plant source is chosen, NP-ScreenR uses LC–MS/MS to rapidly profile its chemical constituents. High-resolution MS generates complex metabolite spectra, which are interpreted using AI-assisted metabolomic workflows. Molecular networking platforms such as GNPS cluster MS/MS spectra by similarity, revealing compound families and enabling early dereplication by linking unknown features to known natural products. In parallel, machine learning models trained on large spectral libraries infer structural features from fragmentation patterns and rank candidate structures. Together, these AI-integrated approaches convert raw MS/MS data into annotated, prioritized molecular features efficiently [72, 102].

### Dereplication and annotation

At this stage, NP-ScreenR focuses on rapid dereplication and annotation to prevent rediscovery of known compounds. Dereplication determines whether detected molecules correspond to previously reported structures. AI-based tools such as SIRIUS combined with CSI:FingerID predict molecular formulas and substructure fingerprints from MS/MS data using machine learning, then match these predictions against natural product databases to generate ranked identifications [72, 103]. In parallel, spectral library matching and molecular networking approaches annotate compounds based on similarity to known metabolites. Additional AI classifiers can assign biosynthetic or scaffold classes, providing contextual information about compound origin and potential bioactivity. As a result, this step yields a curated list of candidates, separating known compounds from potentially novel natural products prioritized for further study [102, 104].

### Candidate structure library

All distinct structures obtained after dereplication, both known compounds and predicted novel ones, are assembled into a candidate structure library representing the chemical space for in silico screening. NP-ScreenR expands this library by integrating public natural product databases, such as large curated repositories of plant-derived compounds, to improve coverage and leverage existing bioactivity annotations. To further explore underrepresented regions of natural product chemical space, AI-based generative models can propose NP-like virtual molecules with realistic structural featuresBy combining experimentally observed compounds with database entries and AI-generated analogs, NP-ScreenR produces a curated and comprehensive library ready for downstream computational bioactivity screening [105, 106].

### NP-aware representation

Before virtual screening, NP-ScreenR converts each molecule into an NP-aware representation suitable for AI analysis. Because natural products have complex scaffolds, stereochemistry, and biosynthetic features, standard descriptors alone are insufficient. Deep learning models such as transformer-based SMILES encoders generate dense embeddings that capture subtle structural patterns, while NP-specific classifiers encode biosynthetic class information directly into the representation. In parallel, graph-based embeddings and classical fingerprints are used to preserve atomic connectivity and topological information. By integrating general chemical embeddings with NP-informed features, NP-ScreenR provides a comprehensive molecular representation that supports accurate downstream bioactivity prediction [90, 104].

### Ligand-based prediction

Using the derived molecular representations, NP-ScreenR applies ligand-based prediction to prioritize candidates with potential anticancer activity. QSAR and machine learning models trained on large bioactivity datasets estimate the likelihood of cytotoxic or anticancer effects based on NP-aware descriptors. In parallel, target-specific ligand-based screening is performed using similarity-based target fishing tools, which compare candidate structures to known ligands to infer probable protein targets. This stage is particularly effective for identifying phytochemicals that resemble known inhibitors of cancer-relevant targets such as kinases, topoisomerases, or signaling proteins. This ligand-based stage efficiently narrows the candidate pool to phytochemicals with promising predicted activities, guiding subsequent structure-based evaluation [90, 107].

### Structure-based screening

As a complement to ligand-based approaches, NP-ScreenR applies structure-based methods to assess how well each candidate interacts with specific cancer targets. Top-ranked phytochemicals are docked in silico into three-dimensional protein structures relevant to cancer biology. When experimental structures are unavailable, high-quality AI-predicted models enable inclusion of otherwise inaccessible targets. Docking algorithms position each compound within the binding site and estimate binding strength, while accounting for the size and complexity typical of natural products. Promising complexes may be further refined using AI-assisted scoring or rapid simulation approaches. This step connects phytochemicals to plausible molecular targets and prioritizes those with strong predicted binding for further study [10, 91].

### Multi-objective ranking

At this stage, NP-ScreenR typically yields several promising phytochemicals with different strengths, such as predicted potency, novelty, or drug-likeness. To prioritize the most viable leads, the framework applies multi-objective ranking that balances key drug discovery criteria. Rather than focusing on activity alone, NP-ScreenR integrates predicted efficacy, target selectivity, ADMET properties, and novelty into a unified evaluation using multi-criteria decision analysis. Algorithms such as Pareto ranking identify candidates that achieve optimal trade-offs. Pareto-based ranking allows flexible prioritization strategies, enabling researchers to emphasize safety, novelty, or potency depending on project goals. This AI-driven prioritization ensures that top hits combine biological promise with practical development potential [108, 109].

### Evidence cards and shortlist

Following ranking, NP-ScreenR produces a shortlist of top-priority phytochemical candidates. To support transparent decision-making, each candidate is summarized in an Evidence Card integrating key predictions, including chemical structure, source information, predicted targets, docking results, and ADMET flags. Where available, supporting literature, analog bioactivity, and omics evidence are also incorporated. These cards provide a concise yet comprehensive rationale for selection, resulting in a well-supported shortlist recommended for experimental validation [90].

### Wet-lab validation and active learning loop

In the final stage, NP-ScreenR transitions from computational prediction to experimental validation. Shortlisted phytochemicals are extracted or synthesized and tested using in vitro and in vivo assays to confirm anticancer activity and target engagement. Crucially, NP-ScreenR incorporates an active learning loop in which experimental outcomes are fed back into the AI models. Both successful hits and false positives are used to retrain prediction and ranking algorithms, improving performance over time. This iterative integration of computation and experimentation enables continuous refinement of the framework and accelerates the discovery of effective plant-derived anticancer agents [101, 110].

## Limitations and future perspectives

### Quality, scarcity and inadequate experimental data integration

AI has become integral to modern drug discovery, supporting key stages such as target identification, compound screening, lead optimization, ADMET prediction and even clinical trial design. Despite these advances, several persistent barriers continue to limit the full potential of AI-driven approaches Chief among these is the challenge of obtaining high-quality, consistent, and representative experimental data. The complex nature of drug–target interactions within biological systems often defies simple binary classification, complicating the training of supervised learning models [111, 112].

Furthermore, the volume of available high-quality data is still limited, especially when compared to the large amount of potentially usable but currently inaccessible or unstructured biological and chemical information. This imbalance restricts the ability of AI models to learn generalizable patterns across diverse chemical spaces. Rodrigues also reported that current datasets inadequately present full diversity of natural products. Numerous plant-derived compounds exhibit unique structural characteristics that are infrequently found in conventional drug-like molecule libraries [98]. Therefore, AI models trained on such datasets often exhibit reduced predictive reliability when applied to phytochemicals.

AI systems also face limitations arising from incomplete, inconsistent, and insufficiently labeled datasets, compounded by the inherent biological complexity of cancer systems and challenges related to model interpretability. Deep learning models are highly-data dependent and without sufficient, high-quality data, their predicative reliability and long-term utility remain constrained [113].

Another major challenge lies in data heterogeneity and inconsistency across studies. As Bender and Cortés-Ciriano noted, the quality of data within natural product databases is often compromised by inconsistencies, missing annotations, and variability in experimental conditions. These issues complicate data integration and significantly hinder the development of robust, generalizable models [114].

Addressing these limitations will require rigorous data standardization, improved curation practices, and enhanced experimental reproducibility. As emphasized by Gaulton et al., the implementation of standardized data protocols is essential to ensure the reliability of AI-driven predictions and to facilitate meaningful comparisons [115, 116]. Only through improved data quality and integration strategies can the full potential of AI in natural product–based drug discovery be realized.

### Data biases of AI models and transparency

Bias in AI can significantly undermine the reliability of its outcomes, often emerging at multiple stages of model development. These biases typically stem from flawed algorithm or issues within the training data and can lead to inequities in application [117]. In the context of drug discovery, AI models are particularly vulnerable to bias due to the limitations of publicly available biological and pharmacological datasets, which tend to overrepresent well-studied compounds. This overemphasis restricts the exploration of novel therapeutic agents and may hinder innovation [118].

Kumar et al. [119] also highlighted that dataset imbalances, biased feature selection, and a general lack of transparency in model design can further compromise both the fairness and accuracy of AI systems. Moreover, inconsistencies between data sources across experimental platforms such as variations in in vitro and in vivo studies results can weaken the reproducibility and trustworthiness of AI-generated predictions [120].

Mitigating these issues requires deliberate efforts to diversify training datasets, standardize data collection protocols, and improve transparency in model development. Such measures are essential for enhancing the robustness, fairness, and credibility of AI applications in phytochemical drug discovery [121].

### Limited model interpretability and Mechanistic insights

The lack of interpretability in AI models continues to be a major barrier to their widespread adoption in drug discovery, as it diminishes confidence int their predictions [122]. Many advanced AI systems are frequently described as “black boxes”, meaning their internal decision-making processes are opaque and difficult to understand. Although methods such as visualization tools and attention mechanisms have been introduced to improve model interpretability, achieving a clear and comprehensive understanding remains a significant hurdle [123].

For example, Zhang et al. developed an AI-based framework to classify meridians by analyzing the topological structures of anti-cancer phytochemicals. While the model demonstrated potential, the authors acknowledged a key limitation including the absence of interpretability analysis related to meridian tropism. They emphasized the importance of integrating chemical profiling with advanced target prediction methods to improve both predictive accuracy and mechanistic insight, highlighting a recurring challenge in AI-based phytochemical research [124]

### Computational resources and expertise constraint

AI and ML hold significant promise for accelerating the discovery of plant-derived anticancer agents by rapidly predicting bioactivity and compound optimization through advanced computational approaches [125]. Computational methods integrated with experimental validation and advanced AI have the potential to revolutionize drug development by optimizing design and improving clinical success rate [126].

However, the development and deployment of AI models are hindered by several technical and resource-related barriers. These include the need for advanced programming expertise, large volumes of well-annotated training data, and access to high-performance computing infrastructure [127]. Ghosh et al. reviewed a range of computational techniques commonly used in phytochemical research including MDS, MM PBSA, ADMET profiling and molecular docking, highlighting both their value and their limitations. They emphasized ongoing challenges such as low accuracy, insufficient datasets, and the critical need for experimental validation to support computational findings [128].

Moreover, Varghese et al. documented that a significant barrier to AI adoption in the field of phytochemicals is the limited availability of user-friendly, accessible software platforms [72]. The lack of accessible tools and computational training among experimental researchers further restricts the widespread implementation of AI-driven discovery pipelines.

### Future prospects

Artificial intelligence (AI) and machine learning have significantly advanced data interpretation and prediction modeling in biomedical research. As these technologies continue to evolve, their integration with multi-omics datasets—including genomics, transcriptomics, proteomics, and metabolomics—through multimodal learning frameworks is expected to further advance disease modeling, diagnosis, and personalized therapy development [129].

In particular cross attention transformer models [130], variational autoencoders [36] and Multiomics factors analysis (MOFA) [131] can be employed to fuse heterogenous omics layers and identify robust biomarkers and therapeutic targets. Such holistic approaches offer a powerful strategy for discovering plant-derived compounds that selectively target cancer-specific pathways [132].

AI has also substantially advanced photochemical research by enabling detailed analysis into biosynthetic pathways and metabolite profiling. One of its most notable contributions lies in the identification of novel therapeutic compounds for cancer treatment [100]. Vision transformers (ViTs) and CNN-transformers hybrids can be applied to spectral and imaging data for identification [133] while graph convolutional Networks (GCN), recurrent neural network (RNN), Message passing Neural Networks (MPNN) and graph attention network (GAT) can improve structure activity relationship model of phytochemicals [124, 134]. In addition, SMILES-based transformers and diffusion models are increasingly used for de novo compound generation and optimization of anticancer activity [135–137].

For predictive screening multitasking deep learning architecture and gradient boosting models (XG Boost) can be implemented for simultaneously activity and ADMET prediction [36, 138]. Docking and binding pose refinement can be enhanced using AI assisted docking framework such as EquiBind and DeepDock thereby improving hit prioritization beyond classical scoring functions [139, 140]. Together, these advances are accelerating AI-driven identification of plant-based compounds for precision oncology.

To fully realize this potential, standardization and sharing of phytochemical-related multi-omics data should be prioritized through international collaborations. Secure and privacy-preserving data sharing may be facilitated through federated learning architectures and blockchain-enabled repositories, enabling cross-institutional collaboration without compromising confidentiality.

Despite its promise, AI-driven phytochemical discovery also raises ethical and regulatory considerations. Bias in data, algorithms, and decision-making processes must be addressed through inclusive dataset design, transparent model development, and rigorous validation strategies [141]. Explainable AI (XAI) techniques, such as SHAP, integrated gradients, and Grad-CAM, are increasingly important for improving transparency and reproducibility [142]. At the same time, the intersection of AI with intellectual property rights and regulatory frameworks is reshaping the governance of drug discovery [143].

Ultimately, multidisciplinary collaboration among computational scientists, biologists, clinicians, legal experts, and regulatory authorities will be essential to overcome both technical and ethical challenges. Through such partnerships, AI enabled phytochemical screening can evolve into a scientifically robust and ethically responsible platform for discovering targeted cancer therapies, ultimately shaping the future of precision oncology [144].

## Conclusion

Artificial intelligence is reshaping the discovery of natural product-derived antitumor agents by enabling faster compound screening, more reliable pharmacological predictions, and more efficient optimization throughout the drug discovery process. By combining machine learning and deep learning approaches with multi-omics data and advanced bioinformatics tools, AI has greatly expanded our ability to explore the chemical diversity of phytochemicals and to identify candidates with genuine anticancer potential.

When integrated with metabolomic, proteomic, and cheminformatic analyses, AI-driven strategies provide a more systematic and informed approach to prioritizing bioactive compounds and understanding their possible mechanisms of action. This represents a meaningful shift away from traditional trial-and-error screening toward more targeted and data-driven phytochemical discovery.

When integrated with metabolomic, proteomic, and cheminformatic analyses, AI-driven strategies provide a more systematic and informed approach to prioritizing bioactive compounds and understanding their possible mechanisms of action. This represents a meaningful shift away from traditional trial-and-error screening toward more targeted and data-driven phytochemical discovery.

Despite these clear advantages, important challenges remain. Issues related to data quality and standardization, limited model interpretability, computational demands, and ethical and regulatory considerations continue to limit the widespread adoption of AI in phytochemical drug discovery. Addressing these challenges will require improved data curation practices, the development of transparent and explainable AI models, and closer collaboration between computational scientists, experimental researchers, and clinicians.

Overall, AI-driven phytochemical screening stands out as both a necessary and promising strategy for the development of new chemotherapeutic agents. As AI technologies mature and become more accessible, their thoughtful integration with experimental validation and clinical insight is likely to play an increasingly important role in advancing precision oncology and natural product–based cancer therapies.

## Data Availability

Not applicable.
